# The genetic legacy of the Manila galleon trade in Mexico

**DOI:** 10.1098/rstb.2020.0419

**Published:** 2022-06-06

**Authors:** Juan Esteban Rodríguez-Rodríguez, Alexander G. Ioannidis, Santiago G. Medina-Muñoz, Carmina Barberena-Jonas, Javier Blanco-Portillo, Consuelo D. Quinto-Cortés, Andrés Moreno-Estrada

**Affiliations:** ^1^ National Laboratory of Genomics for Biodiversity (LANGEBIO), Advanced Genomics Unit (UGA), CINVESTAV, Irapuato, Guanajuato 36824, Mexico; ^2^ Department of Biomedical Data Science, Stanford University, Stanford, CA 94305, USA

**Keywords:** admixture, local ancestry, Mexico, Manila Galleon, Philippines

## Abstract

The population of Mexico has a considerable genetic substructure due to both its pre-Columbian diversity and due to genetic admixture from post-Columbian trans-oceanic migrations. The latter primarily originated in Europe and Africa, but also, to a lesser extent, in Asia. We analyze previously understudied genetic connections between Asia and Mexico to infer the timing and source of this genetic ancestry in Mexico. We identify the predominant origin within Southeast Asia—specifically western Indonesian and non-Negrito Filipino sources—and we date its arrival in Mexico to approximately 13 generations ago (1620 CE). This points to a genetic legacy from the seventeenth century Manila galleon trade between the colonial Spanish Philippines and the Pacific port of Acapulco. Indeed, within Mexico we observe the highest level of this trans-Pacific ancestry in Acapulco, located in the state of Guerrero. This colonial Spanish trade route from East Asia to Europe was centred on Mexico and appears in historical records, but its legacy has been largely ignored. Identities and stories were suppressed due to slavery, assimilation of the immigrants as ‘Indios’ and incomplete historical records. Here we characterize this understudied Mexican ancestry.

This article is part of the theme issue ‘Celebrating 50 years since Lewontin's apportionment of human diversity’.

## 1. Introduction

Population genetics studies have shed light on past historical events and demographic dynamics that in turn are associated with cultural influences [1,2]. Before European contact, the territory of modern-day Mexico was occupied by indigenous peoples (Native Americans), who arrived via one or more founding populations that crossed from Asia to the Americas over what is now the Bering Strait approximately 20 000 years ago [3]. Soon after European contact in the 16th century, the region experienced extensive inter-continental admixture. Europeans and sub-Saharan Africans admixed with local indigenous groups, for example in mining regions of Guanajuato [4]. Around the seventeenth century, admixture began to increase and by the independence of Mexico in the ninteenth century admixed citizens accounted for up to 40% of the population [5]. Several previous studies have investigated the admixture dynamics of these three major colonial-era ancestry components—Native American, European and sub-Saharan African [6,7].

Although Native Americans, Spanish Europeans and sub-Saharan African individuals were the most numerous in Mexico during the colony (as reported by Spanish written records and a census from the seventeenth century [8]), other ethnic groups immigrated to colonial Mexico, in particular groups from Asia. The census estimate from 1646 CE reports a population of 1 712 615 in New Spain, with 74.6% Native Americans, 22.6% admixed and the remainder African (2%) and European (0.8%) minorities [8]. Asians arrived first via the Manila Galleons, ships that conducted the trans-Pacific trade with the Philippines repeatedly between 1565 CE and 1815 CE [9]. The largest period of such migration occurred in the seventeenth century, compensating for the diminished labour force following the indigenous demographic collapse [9]. Some Asian individuals travelled freely to Mexico, but many others were slaves from Manila, where a third of the population was enslaved indigenous Southeast Asian groups [9]. The main New World disembarkation point for these enslaved individuals was the southern Mexican Pacific coastal port of Acapulco in the state of Guerrero. This Asian ancestral contribution to the Mexican population has been overlooked by contemporary genetic studies, which have focused on a tripartite admixture model [10,11]. Asian immigrants in Mexico were often referred to as ‘Indios’ just as Native Americans, and this identity was often embraced by these slaves in order to demand liberation at legal courts [9]. Historical records estimate a total of 40 000–120 000 immigrants from Manila in colonial Mexico [12] and the Spanish recorded that they were particularly numerous in Acapulco, with nearly every Spanish home having at least three, and up to 18, Asian slaves [13]. The genetic legacy of this trans-Pacific trade has not been previously characterized in Mexican genomes.

## 2. Results and discussion

### (a) Beyond the three-way admixture model in Mexico: East Asian ancestry

Genome-wide ancestry proportions were estimated with unsupervised maximum-likelihood clustering (ADMIXTURE) [14] including five continental reference populations and admixed cosmopolitan Mexicans from ten sampled cities in ten different Mexican states. ADMIXTURE clustering at *K* = 5 clusters distinguishes the broad continental components: sub-Saharan African, European, Native American, East Asian and Melanesian (electronic supplementary material, figure S1). This is consistent with the lowest cross validation (CV)-error criterion obtained at *K* = 5 (electronic supplementary material, figure S2), where the five continental references differentiate. Higher *K* values show substructure within continental clusters, mostly among Native American populations. The Austronesian language-speaking groups in Southeast Asia cluster together with the rest of the East Asian populations, so we use the latter as references for local ancestry inference of Southeast Asian and East Asian segments (see §3 for details). This shared clustering of East Asian ancestry with the Austronesian groups of Southeast Asia (*K* = 5 clusters) is supported by the lowest CV-error criterion when running ADMIXTURE (electronic supplementary material, figure S3).

Cluster membership proportions for the admixed Mexican individuals consist mainly of Native American and European components, followed in decreasing order by sub-Saharan African, East Asian and Melanesian. The proportions differ within the Mexican subregions as reported in previous studies [6]. For instance, European ancestry is more prevalent in cosmopolitan samples from northern Mexico, especially in Sonora in the far northwest (62.5% on average with a standard deviation of 9.8%). Meanwhile, Native American ancestry shows higher proportions in southern Mexico, with the highest contribution in Oaxaca (82.9% on average with a standard deviation of 13.7%), according to our ADMIXTURE clustering results at *K* = 5. Sub-Saharan African ancestry reaches 33.4% in individuals from coastal states known for their Afro-Mexican presence [15], namely Veracruz and Guerrero.

**Figure 1.**
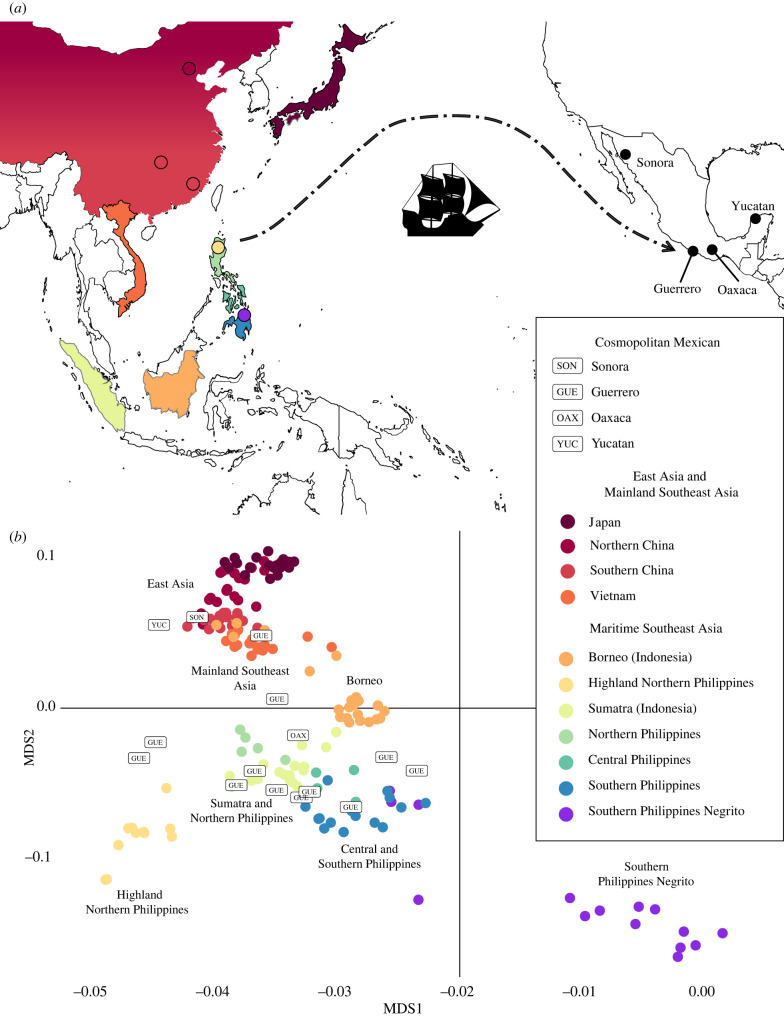
East and Southeast Asian substructure in cosmopolitan Mexican individuals. (*a*) Map showing the sampling locations from East and Southeast Asian populations included in the Asian multidimensional scaling (MDS) shown in panel (*b*). Sampling locations from the Philippines shown in the map as circles represent isolated populations with contrasting genetic profiles. Cosmopolitan Mexican populations with individuals exhibiting more than 5% Asian ancestry are shown in the map with three-letter labels for the state; most of these individuals come from Guerrero. A schematic of the Manila Galleon trade route with an origin in Manila and final destination in Acapulco, Mexico is represented with a dotted arrow. The Manila Galleon sailing path exploited the ocean currents and so formed an arc passing through high northern latitudes before reaching the American coast in northern California and then heading south to its final destination in Acapulco. The Pacific Ocean extent is not shown to scale. (*b*) An MDS shows the East and Southeast Asian reference individuals with filled circles, while cosmopolitan Mexican individuals are plotted with rectangular labels. The colour code in the reference panel matches that of the sampling locations map.

**Figure 2.**
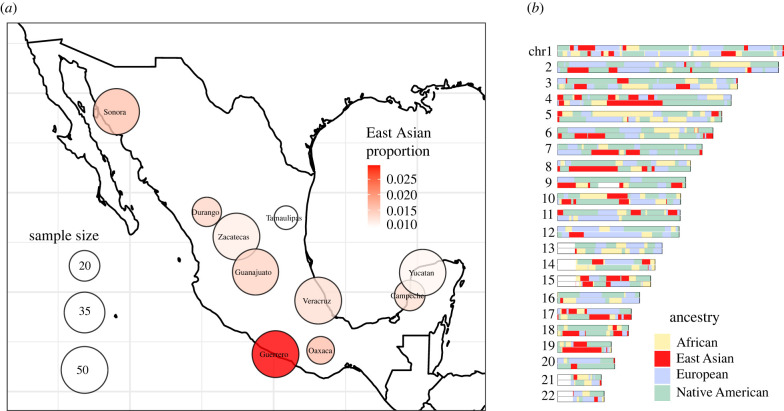
Map of East Asian ancestry average per cosmopolitan Mexican population and local ancestry karyogram of an individual from Guerrero. (*a*) Map of Mexico showing the sampling locations of the populations included in this study. These are labelled according to the Mexican state of each sampling. In most cases the sampling was conducted in the capital city of each state, except for Guerrero where recruitment took place in Acapulco. The colour intensity represents the average East Asian ancestry in each state based on the ADMIXTURE run (*K* = 5), while the node sizes are proportional to the sample size of each state. (*b*) Chromosome painting of an individual from Guerrero used to illustrate the distribution of local ancestry tracts along a genome. The total ancestry proportions in this individual are: East Asian = 15.8%, African = 15.4%, Native American = 40.1% and European = 28.7%.

In this study, we considered a fourth continental origin for the sources that contributed to admixture in Mexico: Asia (figure 1). East Asian and Melanesian ancestries are estimated at less than 5% combined for the majority of cosmopolitan Mexicans. (These two combined genetic components encompass any East Asian, Southeast Asian and Oceanian contributions [16].) However, some individuals in the dataset exhibit more than 5% East Asian and Melanesian ancestry, for instance 12 out of the 50 individuals from the Pacific coastal city of Acapulco, Guerrero, where one individual has over 14% Asian ancestry (electronic supplementary material, table S1). Indeed, Guerrero shows the highest East Asian ancestry proportion of all Mexican populations in this study (figure 2). The high proportions of Asian-derived ancestry in these individuals from Guerrero appear to stem from Asian immigration in the colonial historical period and not to misassigned Native American ancestry since they are not found in the other Mexican states and since they exhibit high variance across Guerrero individuals. For older admixture events, many generations' recombination would equalize these proportions across the population [17,18]. Instead, we observed higher proportions, relative to the other individuals from the same location, indicating that the admixture event was likely recent. Three individuals from Sonora, Oaxaca and Yucatan also show a combined East Asian and Melanesian ancestry of more than 5%. These admixed Mexican individuals had sufficient Asian ancestry to characterize their within-continent origins across Asia in our subsequent analyses.

**Figure 3.**
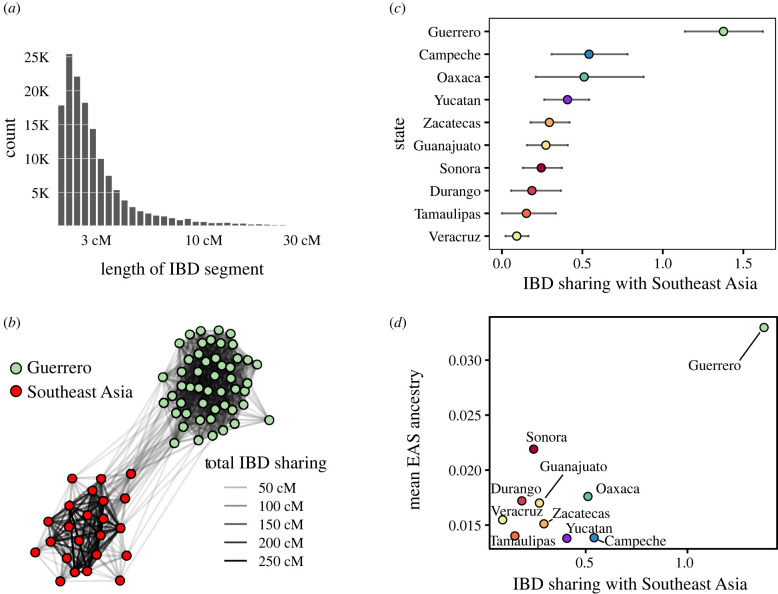
IBD sharing between Southeast Asia and Mexico. (*a*) Length distribution of IBD fragments detected with hap-IBD in the merged data set (Mexican admixed samples and continental reference panels). (*b*) Sub-graph of total IBD sharing in Guerrero and Southeast Asian samples (populations included are those from maritime Southeast Asia detailed in table 1, Arrays C and D). Total IBD sharing is computed by summing the length of IBD segments shared between each pair of individuals. (*c*) Normalized (per individual pair) total IBD sharing of Mexican states with Southeast Asia. The error bars denote the quantiles 0.05 and 0.95 of the bootstrap distribution. To generate the bootstrap distribution, we sample with replacement *N* samples, where *N* is the number of samples in each state, then we compute the mean sum of IBD segments shared per pair of individuals. We repeat this process one thousand times to compute the distribution quantiles. (*d*) Average genome-wide East Asian ancestry versus IBD sharing with Southeast populations for each Mexican state. Guerrero is clearly an outlier, exhibiting both the highest East Asian ancestry cluster membership according to ADMIXTURE, as well as the highest IBD sharing with Southeast Asia (see electronic supplementary material, figure S4 for further details).

An independent line of evidence demonstrating Asian ancestry in Guerrero comes from the identification of genomic segments inherited from common ancestors (identity-by-descent, IBD) between Asian populations and Mexico (figure 3 and electronic supplementary material, figure S4). Guerrero shows higher levels of IBD sharing with Asia than any other Mexican state (figure 3*c*). Moreover, the comparison between East Asian ADMIXTURE proportions and IBD sharing with Southeast Asia shows that Guerrero exhibits an exceptional amount of both compared to the remainder of cosmopolitan Mexicans (figure 3*d*). These results led us to further characterize the subcontinental roots within Asia of these individuals.

**Table 1.** Genotyping arrays used for different analyses. Array A was a pseudo-array constructed by taking the union of the SNPs on two different arrays—Affymetrix 500 K and Illumina 550 K—for samples that were genotyped on both from the Mexican Genome Diversity Project (MGDP) [6]. These included the majority of sampled Mexican states: Sonora, Tamaulipas, Zacatecas, Guanajuato, Veracruz, Guerrero and Yucatan. Those samples that were genotyped only on the Illumina 550 K array are referred to here as Array B and include three states: Durango, Oaxaca and Campeche. Array C included 12 population categories spanning East Asian, Southeast Asian (mainland and maritime) and Oceania genotyped with Affymetrix 6.0: Japan, Northern China, Southern China, Vietnam, Mindanao (Manobo), Negrito from Mindanao, Sumatra (Semende and Besemah), Borneo, Lesser Sunda Islands (Alor, Flores, Roti and Timor), Maluku Islands (Hiri and Ternate), Fiji and Papua New Guinea highlands [22]. Array D included three Filipino sampling locations from [23] re-genotyped with Illumina OmniExpress Bead Chips in [24]: Igorot, Luzon and Visayas. Two whole genome Igorot individuals from the Simons Genome Diversity Project [25] were intersected with this last array dataset.

analysis details	target populations	individuals	markers
**ADMIXTURE**			
**ADMXITURE** analysis	**Cosmopolitan Mexican**^c^**:**	369	509 426
	Sonora, Durango, Tamaulipas, Zacatecas, Guanajuato, Veracruz, Guerrero, Oaxaca, Campeche and Yucatan		
Array A ⋂ Array B			
**TRACTS**			
Asian **Tracts** timings	**Cosmopolitan Mexican**^c^**:**	11^b^	803 636
	Guerrero^a^		
**East Asian MAAS-MDS**			
**RFMix** run	**Cosmopolitan Mexican**^c^**:**	14^a^	803 636
Array A	Sonora^a^, Guerrero^a^ and Yucatan^a^		
**RFMix** run	**Cosmopolitan Mexican**^c^**:**	1^a^	518 409
Array B	Oaxaca^a^		
**RFMix** run	**East and Mainland Southeast Asia**^d^**:**	225	561 339
Array C	Japan, Northern China, Southern China, Vietnam		
	**Philippines**^d^**:**		
	Mindanao and Negrito from Mindanao		
	**Indonesia**^d^**:**		
	Sumatra, Borneo, Lesser Sunda Islands and Maluku Islands		
	**Oceania**^d^**:**		
	Fiji and Papua New Guinea		
**RFMix** run	**Philippines**^e^**:**	22	542 878
Array D	Igorot, Luzon and Visayas		

^a^Only individuals with more than 5% East Asian + Melanesian local ancestry were included in the respective MDS analysis.

^b^Only individuals with more than 4% East Asian + Melanesian global ancestry from ADMIXTURE were included in the respective Tracts dating analysis.

^c^Mexican Genome Diversity Project (MGDP) from [6].

^d^Southeast Asian reference panel from [22].

^e^Filipino population samples from the Southeast Asian reference panel [24] and whole genome Igorot samples from the Simons Genome Diversity Project [25].

### (b) Heterogeneous origins of the Asian ancestry in Mexico

Phasing (separation of an individual's two parental haplotypes) followed by local ancestry inference were applied to the merged dataset (see §3 for details) using SHAPEIT2 [19] and RFMix v. 1.5.4 [20], respectively. These local ancestry results identified the Asian haplotypes in the cosmopolitan Mexican populations, and these were then analysed with ancestry-specific methods including multiple array ancestry specific multidimensional scaling (MAAS-MDS) [21], an MDS designed for analysing samples from several different genotyping arrays simultaneously. Usually, genetic analyses rely on the intersection of genetic markers from all array types used in a study, resulting in extremely limited numbers of common markers remaining. By overcoming this technical difficulty, we can include more populations by being able to combine five different published genotyping array datasets without significant loss of markers (details about these runs are provided in table 1). We tested the accuracy of these local ancestry assignments with MAAS-MDS, and we showed that all four continental ancestries were masked properly for most individuals (electronic supplementary material, figure S5). The algorithm was even able to discern between the pair of most similar continental ancestries: Native American and merged East Asian–Melanesian.

To pinpoint the origin of the Asian component in Mexico, a MAAS-MDS was performed with a reference panel of East Asian, Southeast Asian and Oceanian populations. Only cosmopolitan Mexicans having more than 5% combined East Asian and Melanesian ancestry were included in this analysis, so as to ensure that sufficient sites remained in each individual after masking the other ancestries. This yielded one individual from Sonora, one from Oaxaca, one from Yucatan and 12 from Guerrero. The Sonora and Yucatan individuals clustered near Chinese reference populations, while the Oaxaca and Guerrero individuals clustered within maritime Southeast Asia, with the latter showing a heterogeneous distribution across several source populations on the first two principal components (PCs) (figure 1). No cosmopolitan Mexican sample showed ancestry originating from within the Melanesian islands; therefore, the MAAS-MDS plot was enlarged to focus on the source region in Asia. (The complete MDS plots with and without references from the Melanesian islands are available in the electronic supplementary material, figure S6 and figure S7). Most individuals from Guerrero clustered with maritime Southeast Asia, except for one individual positioned near southern China. Individuals from Guerrero genetically resemble western Indonesian and non-Negrito Filipino populations, specifically those from Sumatra, Mindanao, Visayas and Luzon.

### (c) Admixture timing estimation of Asian gene flow into Mexico

In an admixed population, the length distribution of segments originating from each ancestral source is informative of the time and history of admixture events; for instance, longer segments reflect more recent events for which recombination has had less time to act. Modelling these segment length distributions under different demographic scenarios allows us to identify the more likely history that explains the observed distribution of ancestry tracts. For the Asian-derived segments identified in the Guerrero individuals with over 4% of the ancestry, we performed this fitting using the Tracts algorithm, testing three different models to contrast possible demographic dynamics with the likelihoods given the observed lengths (see §3 for details). We applied a Bayesian Information Criterion (BIC) to compare the fit of these models. The model with a single admixture pulse fit the data best, as compared to the models with continuous migration, i.e. multiple recurrent pulses of the same ancestry across generations (electronic supplementary material, figure S8).

**Figure 4.**
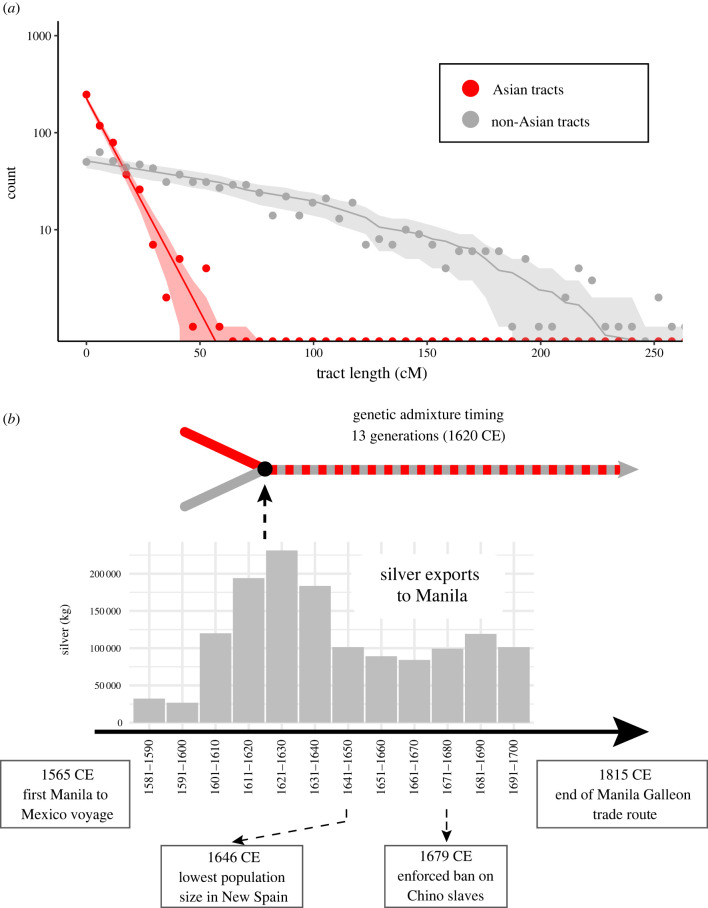
Southeast Asian admixture timing in Acapulco, Guerrero and Manila Galleon trade data. (*a*) Histogram showing the frequency of Asian and non-Asian-derived tracts by length. The expected histogram corresponding to the Tracts model is represented with a solid line, while the shading represents one standard deviation intervals assuming a Poisson distribution. The empirically observed tract length data are shown with points. Asian ancestry tract lengths represent East Asian and Melanesian merged ancestries. Non-Asian tracts consist of merged African, European and Native American ancestries. (*b*) Admixture between Asian and non-Asian ancestry in the Guerrero individuals is represented with an arrow diagram that shows an estimated merging of the ancestries 13 generations ago as determined by a maximum-likelihood analysis. The colour switching along the dotted line of the arrow represents the admixed nature of the resulting population following the merging of the ancestries. Historical silver exports from Mexico on the Manila Galleons [26] are provided as a comparison to genetic dating estimates in a bar plot at bottom. Silver exports from Mexico act as a proxy for voyaging and trade volume, including the purchasing of enslaved persons in Manila by the Galleons and subsequent importation back to Mexico. The dates of the largest registered cargo from Manila to Acapulco and the final enforced ban on ‘Chino’ slaves are indicated. (Online version in colour.)

We found the maximum-likelihood parameters for this single pulse model to be consistent with an Asian admixture event 13 generations ago, or around 1620 CE using a sex-averaged estimate of 30 years per generation (figure 4). This date coincides with the Spanish colonial period and its Manila Galleon slave trade from the Philippines to Mexico, which had a period of major activity from 1565 to 1679 CE [9]. This slave trade route originated in the need for additional labour that arose in colonial Mexico following the demographic collapse of the native populations, and ended when the enslaved Asian source populations, mostly residing in Spanish colonial Asia, were declared indigenous vassals of the crown and thus free. At that time, the Atlantic slave trade from Africa replaced the Pacific route as a source of enslaved humans [9]. The Southeast Asian component stemming from the Manila Galleon trade could have extended to neighbouring coastal Pacific areas of southern Mexico, as appears to be the case for the individual from Oaxaca, although further sampling would be required to investigate this possibility. Although historical records report ‘Chinos’ (as such individuals were called in Mexico) residing predominantly in Guerrero, smaller numbers are also recorded in places such as Colima, Guadalajara, Zacatecas, San Luis Potosi, Veracruz, Puebla, Toluca and, in particular, Mexico City [9]. Thus, we do not rule out the possibility that small amounts of this Asian-derived ancestral component might exist in other Mexican regions, which we do not detect in this study due to insufficient sampling or statistical power.

The East Asian ancestry in the individuals from Sonora and Yucatan, both distant states from Guerrero, has a very different source within Asia and appears to represent post-colonial migration events, perhaps the immigration of Chinese labourers from the Guangdong Province to northern Mexico [27] and the immigration of Korean henequen workers to the Yucatan Peninsula, respectively. These events occurred during and after the Porfiriato Period (between 1880 and 1910 CE) [28]. This hypothesis would need to be tested in the future with more extensive sampling across the country in order to associate these signals more securely with such post-colonial historical events in the far north and south of Mexico.

### (d) Conclusions: a genetic footprint of Asian immigration through the Manila galleon trade

Southeast Asian ancestry was observed in Mexicans from Guerrero, particularly from the Pacific port of Acapulco. This profile suggests a genetic legacy of the Manila Galleon, which used Acapulco as its port of disembarkation in Mexico. Limited historical records indicate that the proximal source of the thousands of ‘Chinos’ who arrived in this city was the Philippines [9]. Our genetic results revealed some Filipino ancestry together with ancestry related to Sumatra in modern Indonesia, then under Muslim Malay rule. Although the Spanish Pacific trade occurred between Manila and Acapulco, this heterogeneity of Asian ancestry in Acapulco can be explained by the multiethnicity of Manila as there was an active slave trade across colonial southeast Asia involving the Portuguese colony of Malacca, the Spanish, and even the Filipino elites that targeted the Muslim-ruled southern islands via the colonial-era concept of ‘just war’. Indeed, Spanish slaves from Sumatra appear in historical records, for instance, Magellan's Malay-speaking slave Henrique, believed to be the first human to circumnavigate the globe. During the Spanish–Moro conflict, sources suggest that soldiers enslaved more than 4000 Muslims between 1599 and 1604 alone [9]. These Muslim Filipinos, named Moro by the Spanish, inhabited the southern Philippines. The genetic affinity of one individual from Guerrero with Mindanao (the southernmost major island in the Philippines) suggests an ancestry perhaps originating in this context. Most of these captives were sold in the Manila slave market [9]. The cultural impact of this migration is still evident in Mexico with the usage of terms of Filipino etymology such as ‘parián’, a word used in Mexico to refer to a market [29]; and the Filipino beverage ‘tuba’, a coconut wine, which was an important industry in the Pacific coast of Mexico and is still traditionally produced in the coastal region of Colima. People from the coast of Guerrero still recognize these Asian cultural influences in their region.

Overall, our results reveal an understudied origin for the historically neglected passage of large numbers of Asian immigrants into Mexican territory during the colonial period. This origin suggests that unraveling the full history of the slave trade within southeast Asia and across the Pacific to Mexico can be pursued through the genetic footprint of present-day admixed populations.

## 3. Material and methods

### (a) Cosmopolitan Mexican dataset

To study the ancestral components of admixed Mexicans, a total of 369 cosmopolitan individuals from ten sampling locations across Mexico were reanalyzed using previously generated and publicly available genotype data [6]. Sampling and genotyping details are provided in the original publication. In brief, we used the Mexican Genome Diversity Project (MGDP) dataset, which consists of seven cosmopolitan populations genotyped on two microarrays, Affymetrix 500 K and Illumina 550 K, and three cosmopolitan populations genotyped on the latter only. The dataset includes 48 individuals from Hermosillo, Sonora; 17 from Ciudad Victoria, Tamaulipas; 19 from Durango City, Durango; 50 from Zacatecas City, Zacatecas; 48 from Guanajuato City, Guanajuato; 50 from Xalapa, Veracruz; 50 from Acapulco, Guerrero; 18 from Oaxaca City, Oaxaca; 20 from Campeche City, Campeche and 49 from Merida, Yucatan. These cities are among the largest and most important from each Mexican state sampled. In all cases, except for Guerrero, recruitment took place in the capital city of the state. To be eligible for inclusion in the study, all four of each individual's grandparents needed to have been born in the state in which they were sampled. All the analyses performed here are focused on these ten cosmopolitan population samples, including the dating of admixture timings, and characterizing Asian substructure differences between Mexican admixed populations.

### (b) Ancestry clustering with ADMIXTURE

The genetic clustering analyses were performed with ADMIXTURE v. 1.3.0 in unsupervised mode. The proportions of five well-differentiated, continental source populations were found for all samples via unsupervised clustering: sub-Saharan African, European, Native American, East Asian and Melanesian. Each continental signal was estimated with an equal number of reference samples when possible. In order to include the largest number of markers, the cosmopolitan Mexican dataset was merged with whole genome reference data from the 1000 genomes consortium dataset [30] and the Human Genome Diversity Project (HGDP) [31]. Individuals from 1000 genomes provided four continental reference panels, while HGDP provided additional Native American individuals plus the Melanesian continental references. 65 Yoruba from Ibadan (YRI) from 1000 genomes represented the sub-Saharan African panel. 65 Iberian from Spain (IBS) from 1000 genomes made the European panel. 27 Peruvian from Lima (PEL) and 2 Mexicans from Los Angeles (MXL) from 1000 genomes and 36 HGDP individuals from the Americas with more than 99% of Native American ancestry made the Native American panel. 33 Kinh Vietnamese (KHV) and 32 Southern Han Chinese (CHS) from 1000 genomes made the East Asian panel. Finally, 16 HGDP individuals from the Papua New Guinea highlands with more than 99% of Australo-Papuan ancestry comprised the Melanesian panel. A total of 509 426 SNPs were considered for an initial ADMIXTURE run before LD pruning, and 175 891 SNPs after linkage disequilibrium (LD) pruning (see electronic supplementary material, figure S1). The merge of Mexican and reference panels was performed with Plink v. 1.90 beta (see https://www.coggenomics.org/plink2/), retaining only the intersection set of markers shared across all panels. In order to consider the genetic structure affinity between East Asian and Southeast Asian populations, an additional ADMIXTURE run was performed including three Filipino populations that are not Negrito, namely, Luzon, Visayas and Igorot individuals. This merged dataset included 118 562 SNPs before LD pruning, and 76 005 SNPs after LD pruning (electronic supplementary material, figure S3).

### (c) IBD analysis with Southeast Asian populations

To compute IBD sharing between Mexicans and Southeast Asian individuals, we used hap-IBD considering segments down to 1.5 cM [32]. Default parameters were used to estimate IBD fragments between these two groups: min-seed = 2.0, max-gap = 1000, min-output = 2.0, min-markers = 100 and min-mac = 2. To estimate the total IBD sharing between Southeast Asia and each state within Mexico, we summed the shared IBD fragments for each pair of individuals and divided by the total number of possible pairwise comparisons (the product of the number of individuals in the two populations). This controls for differences in the number of IBD comparisons run across states. A permutation test was performed to compare the differences in IBD sharing between Asia and Guerrero individuals with the rest of the cosmopolitan Mexican populations (electronic supplementary material, figure S4).

### (d) Phasing with SHAPEIT2 and continental local ancestry assignment with RFMix

The cosmopolitan Mexican and continental references were merged with Plink version 1.90 beta, considering only the intersecting set of markers. Each continental reference panel and admixed Mexican panel was phased separately with SHAPEIT2 and default parameters. Phased haplotypes were given to RFMix v. 1.5.4 [20]. The rephasing step was performed with the PopPhased flag due to the absence of trios and duos in the sample set. Default parameters were used, consisting of 0.2 cM-long windows, eight generations, 100 trees per random forest, zero EM iterations and a minimum of one reference haplotypes per tree node. We considered five continental ancestries as references in the local ancestry pipeline using the same reference individuals from 1000 genomes and HGDP as in the ADMIXTURE analysis. These reference samples were grouped into sub-Saharan African, European, Native American, East Asian and Melanesian.

Because we were investigating events that occurred during the historical period, long after the admixture of East Asian and Melanesian ancestries in prehistoric southeast Asian peoples, after running RFMix we relabelled segments from either of these sources simply as ‘Southeast Asian’ (in the process merging contiguous segments). We then masked all other segments (European, Native American and African) and performed all ancestry-specific analyses only on these ‘Southeast Asian’ ancestry segments. Details for each analysis and run are provided in table 1, specifying number of markers considered and the populations included for the local ancestry calls.

### (e) Asian admixture timing in Guerrero with Tracts

To investigate the dates of Asian admixture in Guerrero, while ignoring the precise labelling of which non-Asian ancestries were counter-party to those admixture events, we applied a general ‘non-Asian’ label to all other ancestry segments called by RFMix above, namely, African, European, Native American. This allowed us to use a simple, robust model with few parameters to focus only on the question at hand, the dates of Asian and non-Asian pulses, without needing to simultaneously investigate subsidiary questions such as the identities of those non-Asian pulses. Using a maximum-likelihood approach, three models were tested using Tracts [33] to find the best fit; these models are hereafter denoted ‘pp’, ‘pc’ and ‘cp’, where p stands for a pulse of incoming migrants and c for continuous migration of a given ancestry across several generations. Different ancestries are thus represented by a single letter, either p or c in this case. The nomenclature of each model dictates the order of the ancestries analysed and the demographic dynamics being tested. The first model involves a single admixture event between both ancestries (pp) and the other two involve one ancestry having a series of multiple recurrent pulses as a model of continuous migration over time. (That is, non-Asian ancestry is modeled as continuous in ‘pc’ and Asian ancestry is modeled as continuous in ‘cp’.) The fits of the predicted and real tract distributions are shown in electronic supplementary material, figure S8. The single pulse model fit the empirical tract distribution best, yielding the highest likelihood (electronic supplementary material, table S3). (Note that all models tested contained the same number of free parameters: two.) Only individuals with combined East Asian and Melanesian ancestry of more than 4% were included in the analysis. Admixture timing results, provided in generations, were converted to dates by considering the date of the sampling in 2010 and assuming generations of 30 years, for the sex-averaged human generation time [34].

### (f) Asian MAAS-MDS with cosmopolitan Mexicans

For the MAAS-MDS analyses, local ancestry was inferred with RFMix using five continental references separately by array. Each array was merged separately with whole-genome sequencing data from 1000 genomes and HGDP for each of the five RFMix runs, allowing for a far higher density of SNPs than a full merge of all five arrays would have, and thus yielding more accurate local ancestry calls. The five continental references were identical to the panel used for genome-wide ancestry clustering. East Asian and Melanesian components were afterwards merged in analyses focusing on localizing the Asian ancestry origins. Local ancestry calls were performed with 803 636 SNPs for Array A, 518 409 SNPs for Array B, 561 339 SNPs for Array C and 542 878 SNPs for Array D (table 1).

We applied a threshold of more than 5% combined East Asian and Melanesian in order for a sample to be considered in the MDS analysis. Samples from cosmopolitan Mexicans included seven populations from Array A and three populations from Array B (table 1). After applying the combined East Asian and Melanesian ancestry threshold filter, only one individual from Sonora, one from Yucatan, twelve from Guerrero and one from Oaxaca were considered in the run. Array C included 12 population categories spanning East Asian, Southeast Asian (mainland and maritime) and Oceania genotyped with Affymetrix 6.0: Japan, Northern China, Southern China, Vietnam, Mindanao (Manobo), Negrito from Mindanao, Sumatra (Semende and Besemah), Borneo, Lesser Sunda Islands (Alor, Flores, Roti and Timor), Maluku Islands (Hiri and Ternate), Fiji and Papua New Guinea highlands [22]. Array D included three Filipino sampling locations from [23] re-genotyped with Illumina OmniExpress Bead Chips in [24]: Igorot, Luzon and Visayas. To this dataset was added two whole-genome Igorot individuals from the Simons Genome Diversity Project [25].

The MAAS-MDS analysis considered the tracts from the combined East Asian and Melanesian ancestry merge, thus masking intercontinental components such as sub-Saharan African, European and Native American, especially in cosmopolitan Mexicans. The analysis was run with average pairwise distances as a dissimilarity measure [35]. All individuals (averaging both haplotypes to create genotype dosage vectors) are plotted as a single point.

## Data Availability

This article has no additional data.
